# Extracellular DNA traps released by acute promyelocytic leukemia cells through autophagy

**DOI:** 10.1038/cddis.2016.186

**Published:** 2016-06-30

**Authors:** R Ma, T Li, M Cao, Y Si, X Wu, L Zhao, Z Yao, Y Zhang, S Fang, R Deng, V A Novakovic, Y Bi, J Kou, B Yu, S Yang, J Wang, J Zhou, J Shi

**Affiliations:** 1 Department of Hematology of the First Hospital, Harbin Medical University, Harbin, China; 2 The Key Laboratory of Myocardial Ischemia, Ministry of Education, Heilongjiang Province, Harbin, China; 3 Department of Research, Brigham and Women's Hospital, VA Boston Healthcare System, and Harvard Medical School, Boston, MA, USA; 4 Department of Cardiology of the First Hospital, Harbin Medical University, Harbin, China; 5 Department of Cardiology of the Second Hospital, Harbin Medical University, Harbin, China; 6 Department of Hematology of the Second Hospital, Harbin Medical University, Harbin, China; 7 Department of Surgery, Brigham and Women's Hospital, VA Boston Healthcare System, and Harvard Medical School, Boston, MA, USA

## Abstract

Acute promyelocytic leukemia (APL) cells exhibit disrupted regulation of cell death and differentiation, and therefore the fate of these leukemic cells is unclear. Here, we provide the first evidence that a small percentage of APL cells undergo a novel cell death pathway by releasing extracellular DNA traps (ETs) in untreated patients. Both APL and NB4 cells stimulated with APL serum had nuclear budding of vesicles filled with chromatin that leaked to the extracellular space when nuclear and cell membranes ruptured. Using immunofluorescence, we found that NB4 cells undergoing ETosis extruded lattice-like structures with a DNA–histone backbone. During all-trans retinoic acid (ATRA)-induced cell differentiation, a subset of NB4 cells underwent ETosis at days 1 and 3 of treatment. The levels of tumor necrosis factor-*α* (TNF-*α*) and interleukin-6 (IL-6) were significantly elevated at 3 days, and combined treatment with TNF-*α* and IL-6 stimulated NB4 cells to release ETs. Furthermore, inhibition of autophagy by pharmacological inhibitors or by small interfering RNA against *Atg7* attenuated LC3 autophagy formation and significantly decreased ET generation. Our results identify a previously unrecognized mechanism for death in promyelocytes and suggest that ATRA may accelerate ET release through increased cytokines and autophagosome formation. Targeting this cellular death pathway in addition to conventional chemotherapy may provide new therapeutic modalities for APL.

Acute promyelocytic leukemia (APL) is characterized by a chromosomal translocation t(15;17), which interrupts the regulation of cell death, differentiation or division.^1^ Drug-induced apoptosis and differentiation/apoptosis are regarded as the main mechanisms in anticancer therapy.^2, 3, 4^ However, a portion of patients undergo relapse partially due to the development of resistance to all-trans retinoic acid (ATRA) and arsenic trioxide (ATO).^5, 6, 7^ In addition, the fate of promyelocytes without chemotherapy is largely unknown. Thus, the mechanisms of cell death in APL need to be explored.

In 2004, Brinkmann *et al.*^8^ discovered a novel cell death program in neutrophils leading to the formation of extracellular traps (ETs) (NETs), termed NETosis, which differs both biochemically and morphologically from apoptosis.^9, 10, 11^ NET formation is involved in inflammatory responses, such as thrombosis and tissue injury.^12, 13, 14, 15^ Although eosinophils, mast cells, monocyte and chronic myelocytic leukemia cell-derived neutrophils have been reported to release ETs,^16, 17, 18, 19, 20, 21^ it remains unclear whether immature granulocytes like APL cells undergo ETosis. Recent reports showed that differentiation of HL-60 cells, a human promyelocytic leukemia cell line, into mature neutrophils with calcium ionophore, ATRA, or dimethyl sulfoxide or exposure to the glycosyltransferase inhibitor tunicamycin (TM) resulted in the formation of NET-like structures.^22, 23, 24, 25^ The nuclei of APL blasts lack lobules and the distribution of chromatin is loose, which are precursors to ET release. In this study, we hypothesized that APL cells are able to form extracellular DNA traps (ETs) and explored conditions under which APL cells are activated. Nuclear alterations occur when cells undergo differentiation; however, the effect of ATRA-induced APL cell differentiation on ET release is poorly understood. Autophagy has been shown to increase differentiation but impede apoptosis of APL cells.^26, 27, 28^ In this regard, autophagy plays an important part in cell regulation, and its role in APL cell ET formation needs to be elucidated. Taken together, the aim of our study was to clarify a novel cell death pathway of APL cells and provide new insights into the pathogenesis and evolution of APL.

## Results

### ET-forming death of APL cells is different from apoptosis

**Figure 1:**
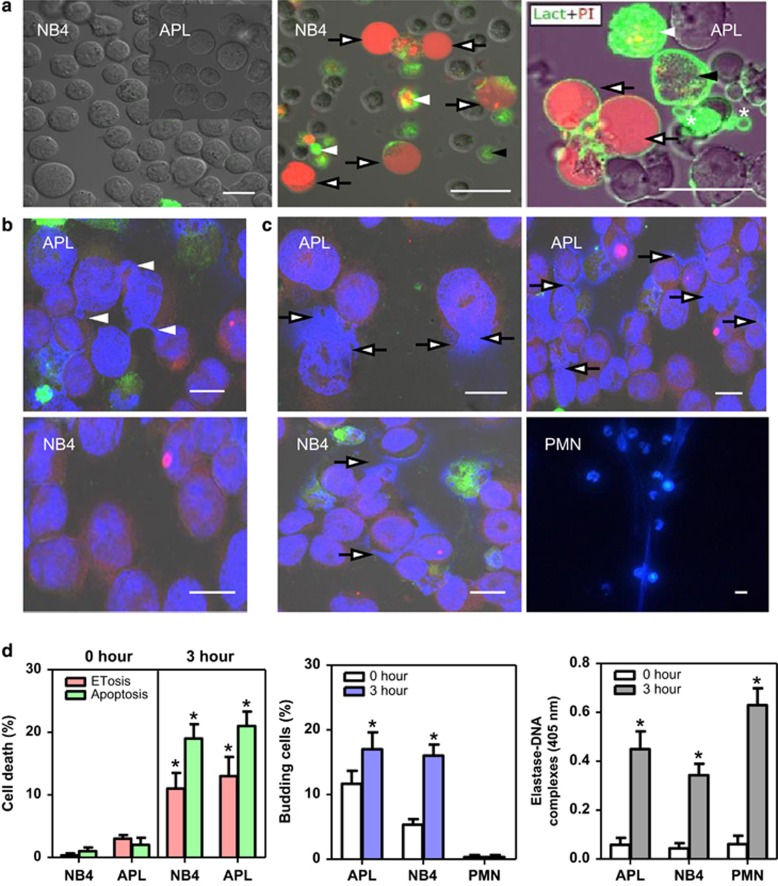
APL cells undergo novel death to form extracellular DNA traps. (**a**) NB4 and APL cells untreated or treated with APL serum for 3 h were stained with lactadherin (green) and PI (red). Confocal microscopy images showed that nuclei lost shape, expanded and filled most of the cytoplasm in treated NB4 cells (middle) and APL cells (right) (arrows). NB4 and APL cells undergoing early apoptosis showed diffuse rim staining by lactadherin but no PI staining (black arrowheads). Cells in late apoptosis had the nuclei that were condensed, fragmented (white arrowheads), and apoptotic bodies (*). Untreated NB4 and APL cells (left) showed little staining with either lactadherin or PI. Cells untreated (**b**) or treated with APL serum for 3 h (**c**) were stained with permeable DAPI (blue). Phosphatidylserine (green) and tissue factor (red) were also stained. Polymorphonuclear leukocytes (PMN) treated with APL serum for 3 h were used as a control. Budding cells (white arrowheads), extracellular DNA traps (arrows). (**d**) Cells undergoing ETosis or apoptosis were counted and analyzed as described in Materials and Methods (*n*=5). Concentration of Elastase–DNA complexes was also measured (*n*=5). All values are means±S.D. *P<0.05 *versus* 0 h. Bars represent 20 *μ*m (**a**) and 15 *μ*m (**b** and **c**)

To know if immature granulocytes can undergo ETosis, cultured NB4 cells and APL cells isolated from patients were incubated with serum from APL patients for 3 h. The phosphatidylserine (PS) probe lactadherin and propidium iodide (PI) were added to distinguish ETosis from apoptosis (Figure 1a). Little fluorescent staining or morphologic alterations were observed in untreated NB4 and APL cells (Figure 1a, left). Some serum-treated NB4 and APL cells were observed undergoing both ETosis and apoptosis (Figure 1a, middle and right). Apoptosis was defined as the presence of membrane blebbing and nuclei fragmentation. The cell membranes of apoptotic cells also stained strongly with lactadherin (green) as a result of the PS expression on the outside of the cell membrane. Cells at later stages of apoptosis after their cell membrane integrity was disrupted stained with PI (red). In contrast, a small subset of NB4 or APL cells underwent a different type of cell death (Figure 1a, middle and right). In these cells, nuclei stained with PI became rounded and nuclear contents rapidly diffused throughout the cell rather than degrading. The PI staining was in the absence of lactadherin membrane staining (green) or visible membrane blebbing. However, due to disruption of the cell membrane, lactadherin did leak into the cell leading to some weak staining (Figure 1a). Permeable 4',6-diamidino-2-phenylindole (DAPI) staining was used to further observe the nuclear changes. We found that fresh APL cells showed a tendency towards deformed nuclei with blebbings (Figure 1b). After treatment with patient serum, some APL and NB4 cells showed disintegrated plasma membranes with their cellular components beginning to leak into the extracellular environment (Figure 1c). In contrast, polymorphonuclear leukocytes (PMN) spat out nuclear structures after treatment with APL serum. About 13% APL cells and 10% NB4 cells underwent ETosis when incubated with APL serum for 3 h, and 21% APL cells and 17% NB4 cells were apoptotic (Figure 1d).

### Nuclear vesicles and breakdown allow the formation of ETs

**Figure 2:**
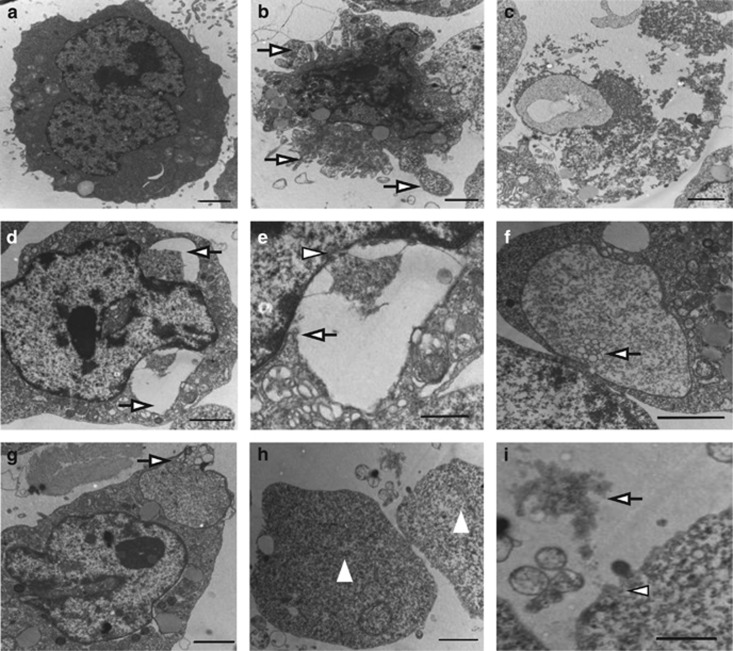
ET-forming death can be distinguished from apoptosis using transmission electron microscopy. (**a**) Normal APL blasts had non-segmented nucleus with intact nuclear membranes. (**b**) APL cells treated with DNR showed characteristic apoptotic morphology, including apoptotic body formation with nuclear condensation and fragmentation (arrows). (**c**) Necrosis in APL cells was characterized by the loss of segregation into euchromatin and heterochromatin while the nuclear envelope remained intact. (**d–i**) APL cells were cultured in APL serum for different time points. (**d** and **e**) After 1 h incubation, inner and outer nuclear membranes separated forming a dilation (arrows) and local magnification showed inner membrane fractured to release nuclear contents into the dilation (arrowhead) while the outer membrane remained intact (arrow). (**f** and **g**) Later (at 2 h incubation), nuclear vesicles merged with granules (arrow) and fused with the plasma membrane to extrude DNA material into extracellular space. (**h**) In end stage (at 3 h incubation), the nuclear envelope ruptured, and DNA filled the cytoplasm leading to cell lysis (triangles). (**i**) Ultimately, the plasma membrane broke (arrowhead) and DNA lattice was released (arrow). Bars represent 2 *μ*m (**a–d, f–h**) and 1 *μ*m (**e** and **i**)

We used transmission electron microscopy (TEM) to investigate the morphological changes leading to ETosis, and distinguish it from apoptosis and necrosis. Untreated APL cells had non-segmented nuclei with intact nuclear membranes (Figure 2a). Apoptosis was induced by incubating APL cells with daunorubicin (DNR) for 24 h.^29^ Apoptotic APL cells showed the classical morphology, including condensation of chromatin and fragmentation of nuclei without rupture of the nuclear envelope, as well as apoptotic body formation (Figure 2b). Necrosis was triggered by subjecting cells to four freeze/thaw cycles.^30^ Necrotic cells were characterized by the loss of nuclei structure and fusion of lobules into a homogeneous mass without segregation into euchromatin and heterochromatin (Figure 2c). APL cells were treated with patients' serum for different time points. When cultured for 1 h, a small percentage of APL cells showed a massive dilation between the inner and outer nuclear membranes referred to as blebbing (Figures 2d and e). The inner membrane was ruptured to release decondensed DNA into this separation (Figure 2e). After 2 h incubation, DNA strands were visible inside vesicles in the cytoplasm. It is worth noting that in the early stages of vesicle formation, the nuclei maintained intact nuclear pore complexes, and breakdown of the nuclear membrane was not evident. The DNA-containing vesicles then merged with granules (Figure 2f) and fused with the plasma membrane to empty their contents and form ETs (Figure 2g). APL cells eventually underwent complete nuclear envelope breakdown, releasing DNA material into the cytoplasm at 3 h incubation (Figure 2h) and then into the extracellular space when the plasma membrane was disrupted (Figure 2i). Thus, cells forming ETs can be distinguished from necrotic cells in that the nuclear membranes remained intact until the latest stage of cell death.

### APL cells release elastase–DNA complexes

**Figure 3:**
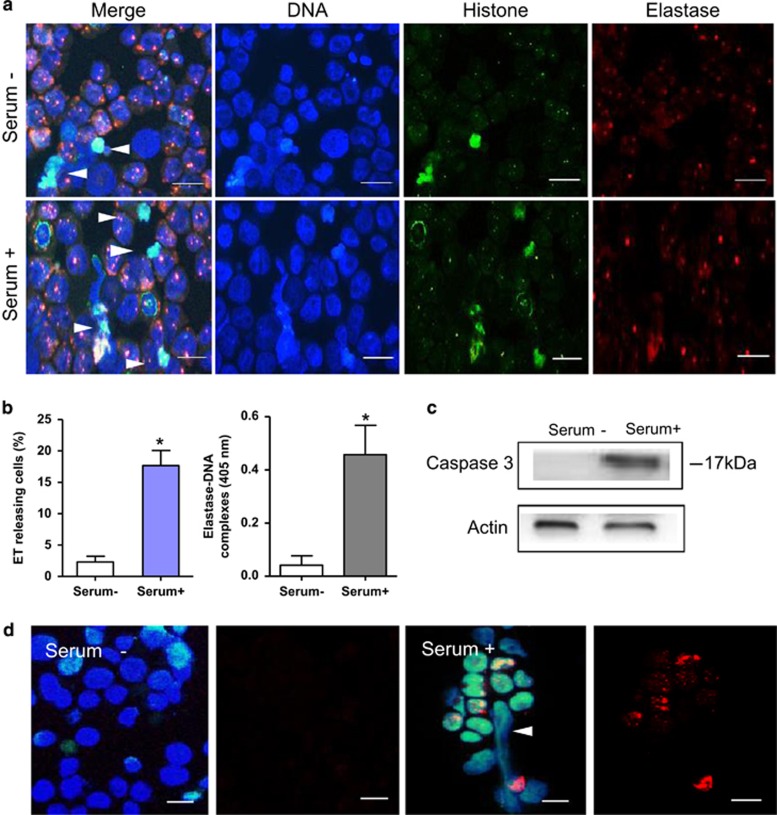
Promyelocytes release elastase–DNA complexes. (**a**) Immunostaining of extracellular DNA traps released by untreated APL cells (upper) or after treatment with APL serum for 3 h (low). Extracellular traps (arrowheads) were characterized by DNA (blue), histone H3 (green) and granule-marker elastase (red). (**b**) Quantification of ETs showed a significant increase in treated APL cells compared with those untreated, consistent with the concentration of elastase–DNA complexes (*n*=3). All values are means±S.D. *P<0.05 *versus* serum−. (**c**) Caspase-3 expression was measured by western blot in APL cells that were untreated or treated with APL serum for 3 h. (**d**) APL cells untreated or treated with APL serum for 3 h were co-stained with DAPI (blue), anti-histone H3 (green) and anti-caspase-3 (red). Immunostaining images of DAPI/Histone/caspase-3 merged (left) or caspase-3 alone (right). APL cells underwent ETosis (arrowhead) with little caspase-3 stain. Bars represent 15 *μ*m (**a** and **d**)

Using immunofluorescence, we were able to identify the components of ETs that were released by APL cells. We found that the granule-marker elastase co-localized with the DNA–histone backbone (Figure 3a). Untreated APL cells from patients released ETs to a lesser extent compared with those treated with APL serum. Based on the recently proposed correlation between circulating nucleosomes, granulocyte activation and ETosis, MPO-DNA or elastase–DNA complexes content in serum was measured as an indirect marker of *in vivo* ET formation.^31, 32^ We found that the elevated ET release in treated APL cells was paralleled by an increased abundance of plasma elastase–DNA complexes (Figure 3b), which was also seen in APL patients in comparison with healthy controls (data not shown). Immunofluorescence and western blot were utilized to measure the apoptosis marker caspase-3 (Figures 3c and d). We found that caspase-3 expression increased in serum-treated APL cells compared with untreated ones, consistent with the finding that more APL cells underwent apoptosis after 3 h of serum treatment (Figure 1d). However, little staining of caspase-3 was seen in ET-releasing APL cells (Figure 3d), providing evidence that ETosis is distinct from apoptosis.

### ATRA induces APL cell differentiation potentiating ET formation

**Figure 4:**
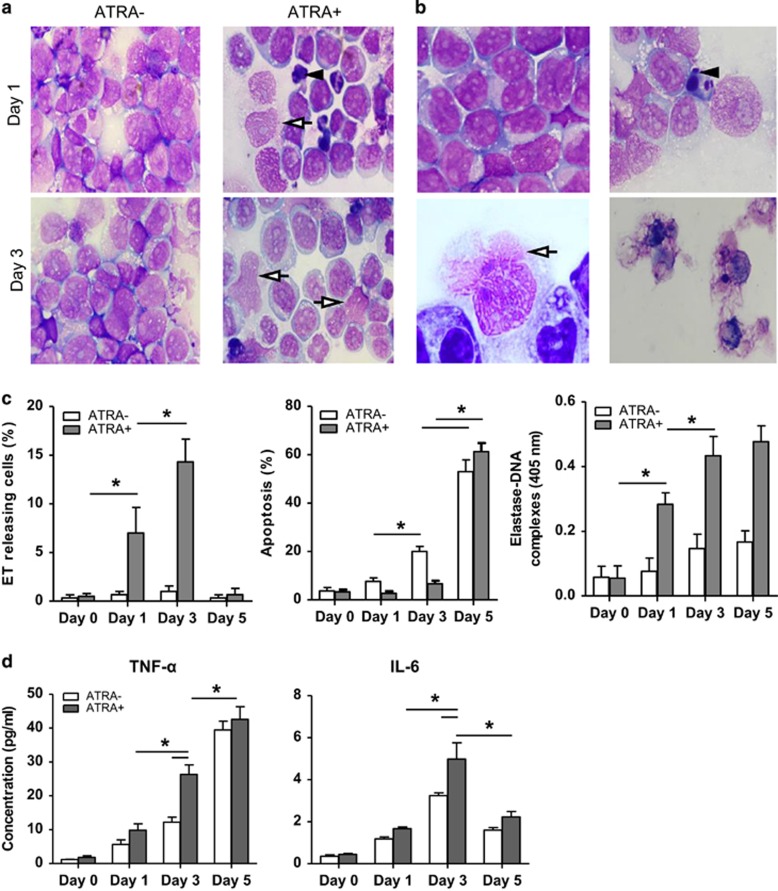
ATRA triggers ET release by NB4 cells during differentiation. NB4 cells were cultured with ATRA (1 *μ*M) for different time points and observed on a light microscope. (**a**) NB4 cells treated for 1 day (upper) and 3 days (lower) were stained with Wright-Giemsa staining, and cell differentiation and nuclear–cytoplasmic ratios were observed. Some NB4 cells died by ETosis (arrows) and apoptosis (arrowhead) during ATRA treatment. Magnification × 100. (**b**) Representative images of cells undergoing apoptosis (upper right, arrowhead) and ETosis (lower left, arrow). After 5 days of treatment, a majority of NB4 cells were undergoing late apoptosis (lower right). Untreated NB4 cells served as a control (upper left). Magnification × 100. (**c**) ATRA-treated NB4 cells were characterized by morphology, and the percentage of ET-releasing cells and apoptotic cells were determined (*n*=5). Concentration of elastase–DNA complexes was also measured (*n*=5). All values are means±S.D. *P<0.05. (**d**) The concentration of TNF-*α* and IL-6 in supernatants was detected with sandwich ELISA using a microplate reader (*n*=5). All values are means±S.D. *P<0.05

NB4 cells showed apparent morphology changes after 1 day of ATRA treatment (Figure 4a). A marked decrease in the nuclear–cytoplasmic ratio was observed after 3 days, and about 12% of NB4 cells had deformed nuclei with the chromatin permeating through the cytoplasm or released from the cells. Apoptotic cells exhibited nucleus concentration and cell shrinkage, which differentiated them from cells undergoing ETosis (Figure 4b). After 5 days, NB4 cells showed cell collapse, indicating late apoptosis. The percentage of cells observed undergoing ETosis peaked at 15% on day 3 (Figure 4c). Because ATRA syndrome is characterized by excessive cytokine release,^33^ we measured tumor necrosis factor-*α* (TNF-*α*) and IL-6 generation in supernatants. Both TNF-*α* and IL-6 were significantly higher in ATRA-treated cells on day 3 (Figure 4d). Furthermore, the levels of TNF-*α* and IL-6 were higher in APL patients compared with healthy subjects (data not shown).

### Autophagy is involved in ET formation

**Figure 5:**
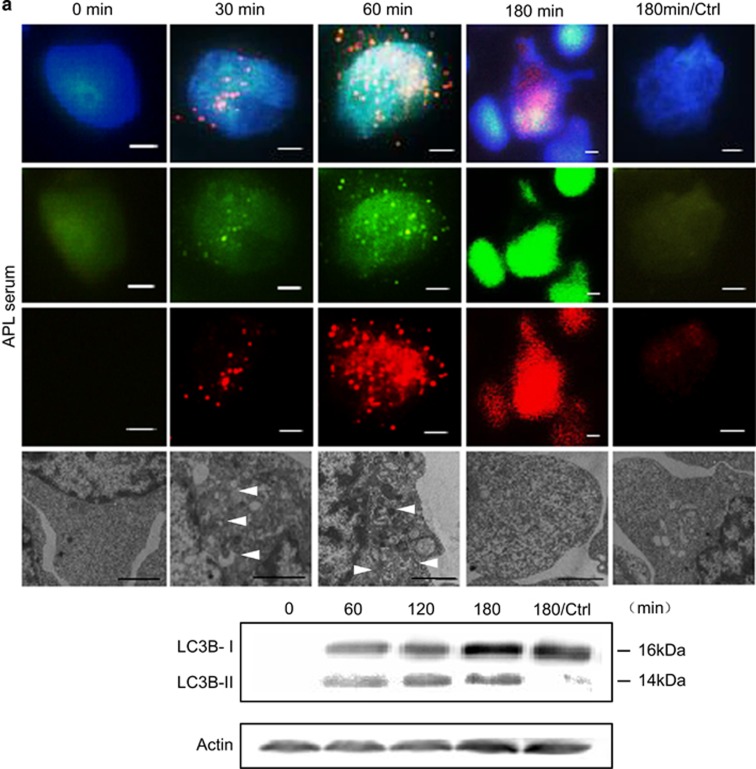

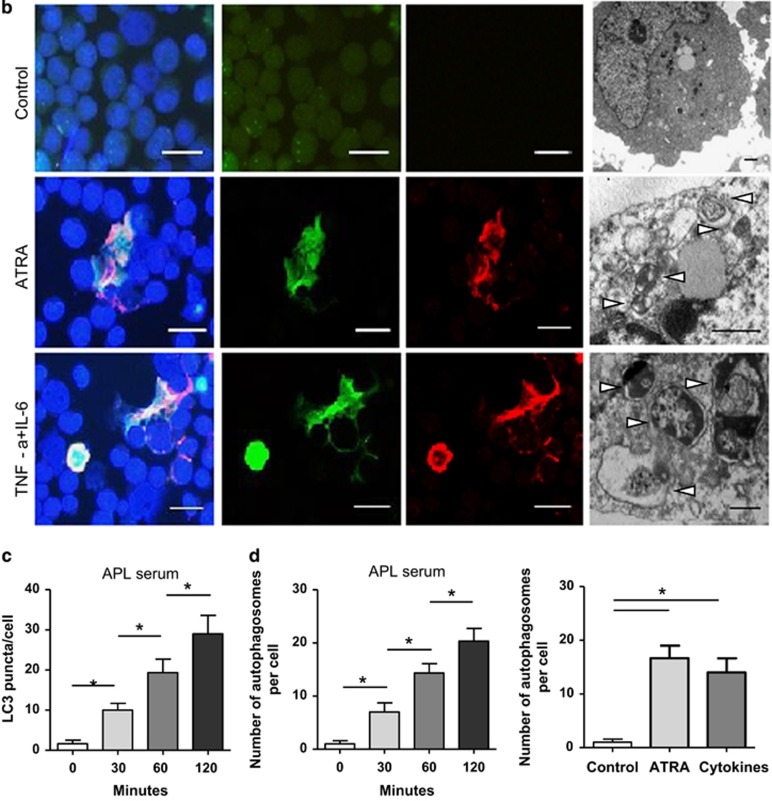
Autophagy is involved in ET formation. (**a**) NB4 cells were incubated with serum from APL patients for different time points and measured by immunofluorescence assays (IF). ET and autophagosome formation were detected by DNA–histone and LC3 positivity, respectively. LC3-coated structures (red) and histones (green) co-localized (yellow) as seen in the merged images. LC3 aggregation accompanied ET release at 3 h. NB4 cells treated with serum from healthy subjects served as controls. LC3I and LC3II expression were measured by western blot. (**b**) NB4 cells were incubated with ATRA for 3 days or stimulated with TNF-*α* and IL-6 for 1 h. Cells were stained with DAPI (blue), anti-histone (green) and anti-LC3 (red). Untreated NB4 cells were used as a control. Transmission electron microscopy (TEM) images further confirmed the presence of double-membrane autophagosomes (arrowheads) during the process of ETosis (**a** and **b**). (**c**) The LC3 puncta per cell in APL serum-treated NB4 cells measured by IF (*n*=3). (**d**) The number of autophagosomes observed by TEM was calculated in APL serum (left panel) and ATRA or cytokine (right panel) treated NB4 cells (*n*=3). All values are means±S.D. *P<0.05. Bars represent 2 (**a**), 15 (**b**, IF) and 1 *μ*m (**b**, TEM)

**Figure 6:**
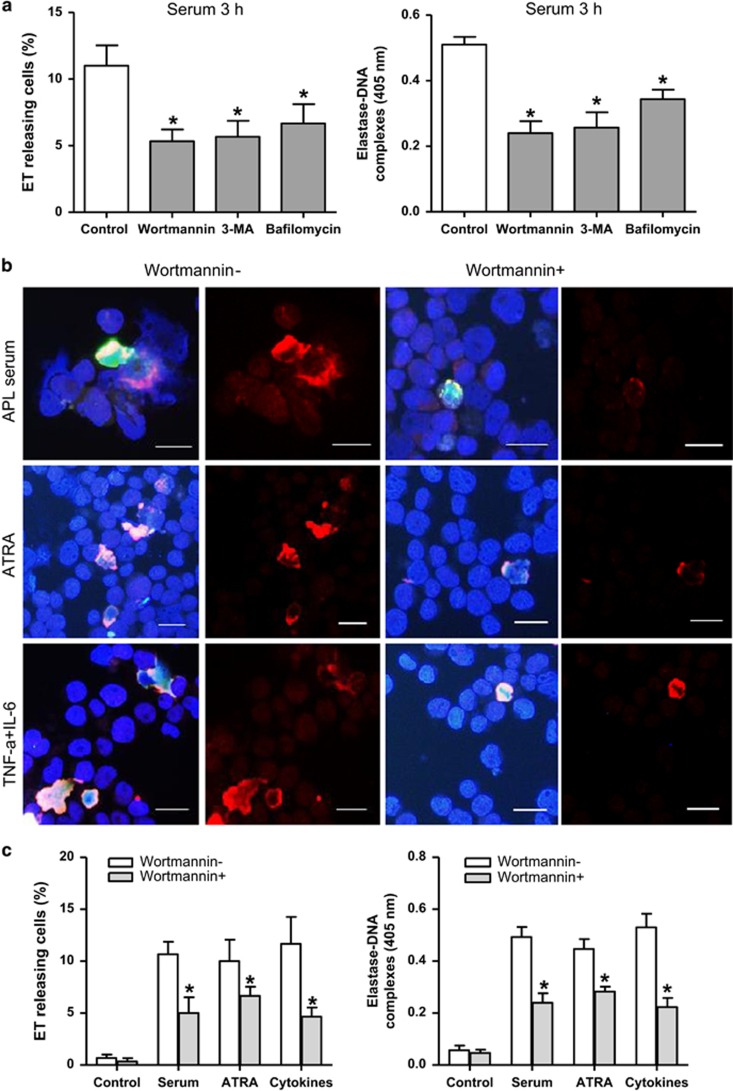
Inhibited autophagy attenuates ETosis. (**a**) NB4 cells were treated with APL serum for 3 h in the presence of various autophagy inhibitors, and the percentage of ET-releasing cells and the concentration of elastase–DNA complexes were measured (*n*=5). All values are means±S.D. *P<0.05 *versus* control. (**b**) NB4 cells were treated with APL serum (3 h), ATRA (3 days) and TNF-*α* and IL-6 (1 h) with or without pretreatment with wortmannin (1 mg/ml). Immunofluorescence images showed inhibition of autophagy by wortmannin attenuated the aggregation of LC3 (red) and resulted in impairment of ET formation (DAPI/Histone/LC3 merged). (**c**) Quantification of the percentage of ET-releasing cells and the concentration of elastase–DNA complexes in different treatments (*n*=5). All values are means±S.D. *P<0.05 *versus* wortmannin (−). Bars represent 15 *μ*m (**b**)

The previously described implication of autophagy in ET release prompted us to investigate whether autophagy contributes to ET formation in APL.^34^ NB4 cells were treated with patients' serum for different time points (Figure 5a) and we found an increase in LC3-positive structures (autophagosomes) and ET formation in a time-dependent fashion. Minimal LC3 aggregation was observed after 30 minutes (min) of stimulation, which was upregulated at 60 min. Of interest, histone colocalization with LC3 was observed in the cytoplasm, indicating that autophagosomes wrapped nucleosomes. LC3 expression (measured by immunofluorescence and western blot) increased in a time-dependent manner. However, LC3 aggregated at the 3 h time point when ET were released making quantitation by immunofluorescence difficult (Figure 5a). ET release was observed at 3 h of stimulation with 13% of NB4 cells releasing DNA threads (Figure 6c). Recent studies suggest that ATRA is closely associated with autophagy in cell differentiation, motivating us to explore whether autophagy contributes to ET formation in ATRA-treated APL cells. NB4 cells were treated with ATRA for 3 days or stimulated with TNF-*α* and IL-6 for 1 h. ET release was significantly increased in ATRA or cytokine-treated NB4 cells in comparison with untreated NB4 cells (Figure 5b). TEM further confirmed that NB4 cells underwent autophagy when stimulated by APL serum or ATRA or cytokines, as indicated by the extensive vacuolization and the formation of typical autophagosomes, defined at the ultrastructural levels by a double membrane (Figures 5a and b). The increased numbers of autophagosomes observed by TEM in APL serum or ATRA or cytokine-treated NB4 cells were consistent with enhanced LC3 staining (Figure 5d). These results indicate that autophagy does occur when APL cells undergoing ETosis.

### Blocking autophagy attenuates ET release by NB4 cells

**Figure 7:**
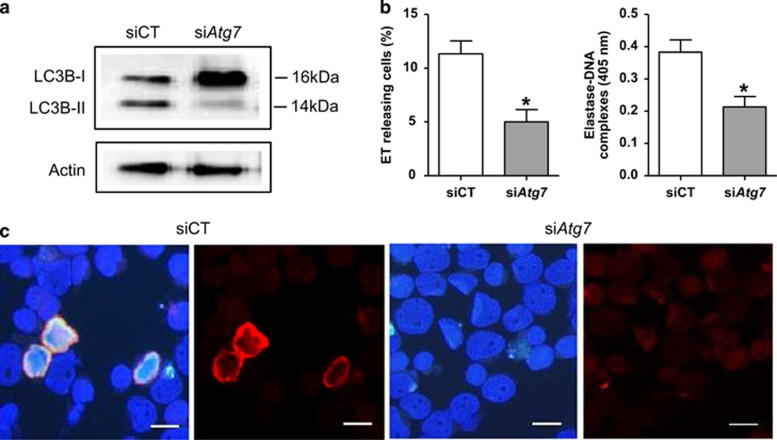
Inhibition of autophagy by knocking down *Atg7* decreased ET release. NB4 cells were transiently transfected with *Atg7* siRNA (si*Atg7*) at a concentration of 100 nM and scrambled siRNA (scr) was used as a negative control (siCT). Seventy-two hours after transfection, cells were treated with APL serum for 3 h. (**a**) The levels of LC3B-I and LC3B-II were detected by western blotting. (**b**) Quantification of the percentage of ET-releasing cells and the concentration of elastase–DNA complexes (*n*=5). All values are means±S.D. *P<0.05 *versus* control. (**c**) Immunofluorescence images showed inhibition of autophagy by knocking down *Atg7* reduced the aggregation of LC3 (red) and ET formation (DAPI/Histone/LC3 merged). Bars represent 15 *μ*m

NB4 cells were cultured with APL serum for 3 h in the presence of various autophagy inhibitors (5′-(4-fluorosulfonylbenzoyl) adenosine hydrochloride (wortmannin), 3-methyladenine (3-MA) or bafilomycin) resulting in significantly decreased ET release (Figure 6a). For further experiments, NB4 cells were treated with APL serum (3 h) or ATRA (3 days) or TNF-*α* and IL-6 (1 h) with or without wortmannin, which blocks autophagic flux. In all three groups, wortmannin inhibited both LC3 aggregation and the ET release (Figure 6b). Elastase–DNA complexes in supernatants also decreased with wortmannin treatment (Figure 6c). Furthermore, we knocked down autophagy-related gene *Atg7* using small interfering RNA (siRNA) to confirm the role of autophagy in ETosis in APL.^35^ As shown in Figure 7a, inhibition of autophagy nearly abolished APL serum-induced autophagy as the markedly decreased conversion of LC3B-I to LC3B-II. Knocking down of *Atg7* (supplementary figure) resulted in significantly reduced ET release and LC3 expression by immunofluorescence (Figure 7c) and elastase–DNA complexes generation (Figure 7b). Combined with results in Figure 5, we suggest that APL serum/ATRA/cytokine-induced ET release by NB4 cells may proceed in an autophagy-dependent manner.

## Discussion

In this study, we have shown for the first time that APL blasts release intact chromatin into the extracellular space leading to ET formation. The changes follow a particular pattern that is initiated by the dilation of the inner and outer nuclear membranes. After that, ETosis is characterized by nuclear vesicle formation and exocytosis to extrude DNA strands. Later, APL cells undergo nuclear envelope and cell membrane breakdown, allowing the DNA traps to leak out. It is worth noting that ET-forming death is distinct from apoptosis and necrosis in morphology, suggesting a novel cell death pathway. More importantly, the vesicles used by APL cells to deliver DNA traps are distinct from those used by PMNs when spitting out DNA strands, and the dynamics of these mechanisms need to be further studied. The main components of ETs are DNA and histone which are procoagulant substances.^36, 37^ Recent studies have implicated ETs in thrombotic events in acute myocardial infarction, venous thromboembolism, tumor, etc.^38, 39, 40, 41^ The role of ETs in the pathogenesis and coagulopathy of APL needs further study.

ATRA is acknowledged to induce cell differentiation and is widely used in APL patients. However, we report for the first time that about 10% of cells undergo ETosis instead of differentiation during ATRA treatment. In a time-dependent manner, TNF-*α* and IL-6 concentrations are increased in ATRA-treated APL cells. Additionally, combined treatment with TNF-*α* and IL-6 is capable of stimulating APL cells to release ETs. Given these results, we speculate that ATRA both accelerates cell differentiation and induces cells to release more cytokines, such as TNF-*α* and IL-6. TNF-*α* and IL-6 are potent stimuli of granulocytes.^42, 43^ Thus ATRA may promote APL cells to generate ETs through TNF-*α* and IL-6 activating pathways. Furthermore, recent studies have suggested that ATRA promotes differentiation of APL cells by inducing degradation of the PML-RAR*α* oncoprotein. Whether the incomplete degradation of PML-RAR*α* oncoprotein or another ATRA-induced signal pathway contributes to ETosis is largely unknown and urgently needs studying. This could provide important clues for ATRA-associated drug resistance and new therapeutic targets.

Autophagy is a well-conserved, essential, intracellular degradation process and occurs when cells respond to stress or undergo death.^44, 45, 46^ Recent studies demonstrate that parts of the nucleus can be specifically degraded by an autophagic process termed nucleophagy.^47^ In this study, we find that in the early stage of ETosis, nuclear material are sequestered and packed in vesicles. Colocalization of DNA–histone and LC3 is also seen, suggesting that nucleophagy may be the cause of the nuclear vacuolization that initiates ETosis. However, the nuclear vesicles mix with granules and are released to the extracellular space, and ultimately the autophagosomes merge when ET form. This is consistent with the morphology of cell lysis in the end stage of ET release and different from nucleophagy. Further, inhibiting autophagy attenuates ET formation in APL serum, ATRA or cytokine-treated APL cells. These results indicate that autophagic sequestration of part of the nucleus may be the initiation of ETosis and nuclear degradation by autophagy may contribute to the elimination of abnormal oncogenes.^48, 49^ Furthermore, the molecular mechanisms involved in nuclear autophagy in APL cells need to be further studied.

APL has evolved from being a deadly to a highly curable disease, due to combined treatments of ATRA with ATO or chemotherapy. However, drug resistance and recurrent disease are still critical problems in APL. As a different cell death pathway, ETosis is considered as important as apoptosis for its widespread existence in various physiologic and pathologic conditions. In this regard, ETosis as well as apoptosis are inherent self-regulation pathways in APL. If applied in clinical treatment, induction of APL cell ETosis could be an alternative to killing tumor cells. Our study provides new insights into the regulatory mechanism of cell death, further insights into the evolution of promyelocytes and pathogenesis, and potential points of molecular intervention strategies in APL patients. Our results, showing the release of intact chromatin decorated with cytoplasmic proteins into the extracellular space by APL cells, are unprecedented and demonstrate a novel cell death pathway that warrants further investigation.

## Materials and Methods

### Patients

Venous peripheral blood and bone marrow were obtained from 16 newly diagnosed APL patients admitted to the First and Second Affiliated Hospital of Harbin Medical University between May 2014 and February 2015. Informed written consent was obtained from every patient or their relatives. The diagnosis was based on clinical data, morphology, cytochemistry, immunology, cytogenetics and molecular biology.^29^ Cytogenetic analysis indicated the presence of t(15;17) translocation and PML/RAR*α* fusion gene in all cases. The main characteristics of the patients are reported in Table 1. This study was approved by Ethics Committee of Harbin Medical University and conducted in accordance with the Declaration of Helsinki.

### Reagents

RPMI 1640 medium and fetal bovine serum (FBS) were obtained from Gibco (Grand Island, NY, USA). Ficoll-Hypaque, poly-d-lysine, bovine serum albumin (BSA), DAPI, DNR, ATRA, TNF-*α*, IL-6, wortmannin, 3-MA and bafilomycin were from Sigma-Aldrich (St. Louis, MO, USA). PI was from Shanghai Dobio Co., Ltd (Shanghai, China).

FITC-lactadherin was obtained from Haematologic Technologies Inc. (Burlington, VT, USA). Rabbit anti-histone 3-Alexa Fluor 488 mAb (ab154206), rabbit anti-neutrophil elastase (NE) mAb (ab131260) and rabbit anti-caspase-3 (ab32042), rabbit anti-LC3B (ab48394), rabbit anti-*Atg7* (ab52472) were from Abcam (Cambridge, MA, USA). Rabbit anti-LC3*α*/*β* polyclonal Ab (sc-292354) was obtained from Santa Cruz Biotechanology Inc. (Santa Cruz, CA, USA). Goat anti-rabbit Cy3 antibody (Invitrogen, Carlsbad, CA, USA) was utilized as a secondary antibody.

### Cell and cell culture

Freshly isolated APL blasts were obtained from bone marrow specimens by centrifugation through Ficoll-Hypaque and were used for experiments immediately. In some cases, these cells (5 × 10^5^/ml) were propagated in complete RPMI 1640 medium supplemented with 20% FBS, 2 mM l-glutamine and 1% penicillin–streptomycin solution at 37 °C in a 5% CO_2_ humidified atmosphere. Human APL NB4 cells were maintained under the same conditions except that 10% FBS was used. In some cases, NB4 cells were treated with 1 *μ*M ATRA for differentiation. Polymorphonuclear leukocytes were obtained from peripheral venous blood of healthy subjects using density gradient separation and resuspended in RPMI medium for immediate experiments.

### Stimulation and inhibition of NB4 cells

Peripheral venous blood was collected and serum was immediately isolated by centrifugation at 4 °C at 1400  × *g* for 15 min.^50^ Serum was stored at −80 °C till used. NB4/APL cells or neutrophils were incubated with sera at a final concentration of 20% at 37 °C.^50, 51^ In some cases, NB4 cells were stimulated with TNF-*α* (100 ng/ml) and IL-6 (100 ng/ml) for 1 h.^54^ For inhibition assays, the autophagy inhibitor wortmannin (1 mg/ml), 3-MA (1 mg/ml) or bafilomycin (1 mg/ml) was incubated with NB4 cells 30 min before stimulation.

### Confocal microscopy

To characterize cell death, the smear of APL or NB4 cells (5 × 10^5^) were incubated with DAPI (100 ng/ml)^20, 51^ or the indicated concentration of PI and FITC-labeled lactadherin.^53^ Cells were washed to remove unbound proteins and analyzed immediately on a Zeiss LSM 510 Meta confocal microscope (Carl Zeiss Jena GmbH, Jena, Germany). The samples were excited with 488 nm emission line of a krypton-argon laser, and narrow bandpass filters were utilized to restrict emission wavelength overlap. ETosis was distinguished by cells whose nuclei stained with PI became rounded and nuclear contents diffused throughout the cell or by cells stained DAPI with budding and extruding DNA. Apoptosis was defined as the presence of membrane blebbing and nuclei fragmentation. Cells were counted from six random fields in triplicate wells for each condition and expressed as the percentage of total number of cells in the field.^20^

### Induction of cell apoptosis and necrosis

APL or NB4 cells were incubated with 1 *μ*M DNR for up to 24 h to induce apoptosis.^29^ Necrosis was elicited in APL cells with four cycles of freezing (liquid nitrogen) and thawing (37 °C), leading to complete fragmentation of the cells.^30^

### Transmission electron microscopy

For TEM experiments, cells were collected and double fixed in 2.5% glutaraldehyde and 1% OsO_4_. After dehydration and embedding, ultrathin sections were prepared with a Reichert-Jung Ultracut Ultramicrotome (Leica, Vienna, Austria). Images were observed under a H7650 transmission electron microscope (Hitachi Ltd, Tokyo, Japan).

### Immunofluorescence assays

APL or NB4 cells were seeded on glass coverslips with poly-lysine in a 24-well culture plate, treated with stimuli or left unstimulated. Cells were fixed with 4% PFA, blocked (2% BSA) and incubated with primary anti-histone coupled with Alexa Fluor 488 (1:100); anti-LC3*α*/*β* (1:50), anti-caspase-3 (1:50) and anti-NE (1:100) were detected with secondary antibodies coupled to Cy3 (1:50). For DNA detection, DAPI (100 ng/ml) was used. Specimens were mounted and analyzed on a fluorescence microscope (Leica, DM400B, Wetzlar, Germany). For quantification, ETs were counted from six different fields in triplicate wells for each condition and expressed as the percentage of ET-forming cells per total number of cells in the field.^20^

### Western blotting

Whole-cell lysates were resolved on a denaturing 10% SDS-PAGE gel and subsequently transferred to polyvinylidene fluoride membranes via semidry transfer. After blocking the membrane at room temperature with 5% skim milk for 3 h, the membrane was incubated overnight at 4 °C with anti-caspase-3 (1:500) or anti-LC3II (1:1000) antibodies. After incubation with peroxidase-conjugated secondary antibodies at a dilution of 1:2000 for 1 h and washed three times with PBS, the signals were visualized using enhanced chemiluminescence.^26^

### Determination DNA–elastase and cytokine levels in plasma

Plasma from healthy subjects and APL patients were prepared as previously described.^54^ Briefly, blood was collected and plasma was isolated by centrifuging blood for 5 min at 2300 ×  *g*. Plasma supernatant was carefully removed and centrifuged again for 5 min at 2300 ×  *g* to remove any remaining blood cells. Plasma was stored at −80 °C until analysis. Elastase–DNA complexes were detected using a capture ELISA.^31, 32^ Briefly, diluted plasma were added to high-binding 96-well ELISA microplates (Greiner Bio-One, Frickenhausen, Germany) pretreated with capture anti-NE (1:2000; Calbiochem, Darmstadt, Germany, rabbit) antibody and blocked with 1% BSA/0.1% human serum albumin/PBS for 1–2 h. After overnight incubation and three washes with Tween20 in PBS, secondary peroxidase-labeled of anti-DNA monoclonal antibody (Roche, Indianapolis, IN, USA; Cat. No:11774425001) was added for 30–60 min at RT according to the manufacturer's instructions. The samples were washed three times with PBS per well and the peroxidase substrate (ABTS) of the kit (Roche; Cat. No:11774425001) was added. The absorbance at 405 nm wavelength was measured using the Tecan microplate reader (Tecan Infinite M200) after 40 min incubation at 37 °C in the dark. TNF-*α* and IL-6 in plasma or culture supernanants were measured with a Human TNF-*α* ELISA kit and Human IL-6 ELISA kit (Roche) according to the manufacturer's instructions.^52^

### siRNA transfection

NB4 cells were transiently transfected with *Atg7* siRNA (no. 6604; Cell Signaling Technology, Danvers, MA, USA) at a concentration of 100 nM using Lipofectamine RNAiMAX Transfection Reagent (Life Technology, Grand Island, NY, USA), according to the manufacturer's instruction (RNAiMAX Reverse Transfections Lipofectamine). The cells were also treated with a scrambled siRNA (no. 6568; Cell Signaling Technology) as a negative control. Seventy-two hours after transfection, cells were treated with the APL serum for 3 h. The silencing efficiency of *Atg7* was determined by western blot of LC3 expression.^35^

### Quantitative real-time PCR

The mRNA levels of *Atg7* was measured as previously described.^55^ Briefly, total RNA was extracted using Trizol Reagent (Invitrogen) and reverse transcribed into cDNA using first strand cDNA synthesis kit (Roche Diagnostics) as per the manufacturer's instructions. Real-time PCR was performed in an ABI PRISM 5700 Sequence Detector System (Applied Biosystems) using the SYBR Green detection protocol as outlined by the manufacturer. A gene-specific primer for *Atg7* was designed using the Primer Express software (Applied Biosystems). Quantitative normalization of the cDNA in each sample was performed using the *GAPDH* gene as an internal control. Real-time PCR assays were performed in duplicate for each sample, and the mean value was used for the calculation of mRNA expression levels.

### Statistical analysis

Numerical variables were tested for normal distribution with the Kolmogorov–Smirnov test. Data are expressed as mean±S.D. for at least three replicates, and statistical analysis was made by *t*-test or ANOVA as appropriate.

## Figures and Tables

**Table 1:** APL patients' characteristics

**No**	**Sex/age**	**Diagnosis**	**Blasts**	**WBC**	**Plts**	**Hb**
			**(BM%)**	**(× 10**^**9**^**/l)**	**(× 10**^**9**^**/l)**	**(g/l)**
1	F/48	M3/bcr1	78	17.9	26	57
2	F/34	M3/bcr1	61	29.3	27.4	89
3	M/37	M3/bcr3	96	1.54	11.3	64.5
4	M/66	M3/bcr1	75	2.59	5.39	44
5	F/29	M3/bcr1	94	19.6	31	19
6	F/64	M3/bcr1	72	2.9	86	102
7	F/24	M3/bcr2	88	32.1	19.8	65
8	M/51	M3/bcr1	98	4.5	53	60
9	M/62	M3/bcr1	57	5.6	62	100
10	F/75	M3/bcr2	96	2.2	170	79
11	M/26	M3/bcr1	69	0.8	10.8	103
12	F/25	M3/bcr1	84	12.6	148	89
13	M/45	M3/bcr3	90	0.4	27	75.2
14	M/76	M3/bcr2	85	1.7	15	76
15	M/18	M3/bcr1	73	145.2	79	87
16	M/33	M3/bcr1	81	0.9	10	92
Ref. range			0–0.4	4–10	100–300	110–170

The main clinical and laboratory features of 16 newly diagnosed APL patients at the moment of bone marrow (BM) aspiration were reported. Blasts, promyelocytes+blasts; WBC, white blood cell; Plts, platelets; Hb, hemoglobin; bcr, breakpoint cluster region (bcr1=intron 6, bcr2=exon 6, bcr3=intron 3).

## Abbreviations

APL: acute promyelocytic leukemia
ATO: arsenic trioxide
ATRA: all-*trans* retinoic acid
ETs: extracellular DNA traps
GAPDH: glyceraldehyde-3-phosphate dehydrogenase
LC3: microtubule-associated protein 1 light chain 3
IL: interleukin
PI: propidium iodide
NE: neutrophil elastase
PMN: polymorphonuclear neutrophil
PS: phosphatidylserine
TEM: transmission electron microscopy
TM: tunicamycin
TNF: tumor necrosis factor
