# Supplementary material for: Extracellular DNA traps released by acute promyelocytic leukemia cells through autophagy
Source: Cell Death Dis. 2016 Jun 30;7(6):e2283–. doi: 10.1038/cddis.2016.186 (PMC5108337; doi:10.1038/cddis.2016.186)
Supplement: Supplementary Figure [file cddis2016186x1.pdf]

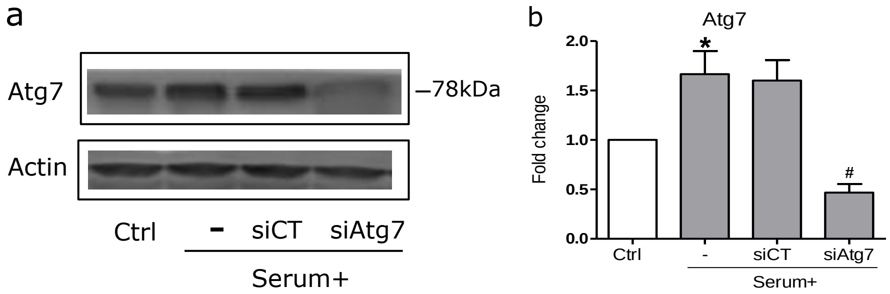

Supplemental Figure. The mRNA and protein expression of Atg7. NB4 cells were transiently transfected with Atg7 siRNA (siAtg7) at a concentration of 100 nM and scrambled siRNA (scr) was used as a negative control (siCT). Seventy-two hours after transfection, cells were treated with APL serum for 3 h. (a) The levels of Atg7 were detected by Western blotting. (b) Quantification of mRNA levels of Atg7 in NB4 cells were detected by qRT-PCR. All values are means ± SD. * $P<0.05$ versus control; # $P<0.05$ versus siCT.
